# Cognitive exertion affects the appraisal of one’s own and other people’s pain

**DOI:** 10.1038/s41598-023-35103-w

**Published:** 2023-05-19

**Authors:** Laura Riontino, Raphaël Fournier, Alexandra Lapteva, Nicolas Silvestrini, Sophie Schwartz, Corrado Corradi-Dell’Acqua

**Affiliations:** 1 Department of Psychology, Faculty of Psychology and Educational Sciences, University of Geneva, Geneva, Switzerland; 2 Department of Neuroscience, Faculty of Medicine, University of Geneva, Geneva, Switzerland; 3 University of Geneva – Campus Biotech, Chemin Des Mines 9, 1211 Geneva, Switzerland; 4 University of Bern, Bern, Switzerland; 5 Swiss Center for Affective Sciences, University of Geneva, Geneva, Switzerland; 6 Geneva Neuroscience Center, University of Geneva, Geneva, Switzerland

**Keywords:** Neuroscience, Psychology

## Abstract

Correctly evaluating others’ pain is a crucial prosocial ability. In both clinical and private settings, caregivers assess their other people’s pain, sometimes under the effect of poor sleep and high workload and fatigue. However, the effect played by such cognitive strain in the appraisal of others’ pain remains unclear. Fifty participants underwent one of two demanding tasks, involving either working memory (Experiment 1: N-Back task) or cognitive interference (Experiment 2: Stroop task). After each task, participants were exposed to painful laser stimulations at three intensity levels (low, medium, high), or video-clips of patients experiencing three intensity levels of pain (low, medium, high). Participants rated the intensity of each pain event on a visual analogue scale. We found that the two tasks influenced rating of both one’s own and others’ pain, by decreasing the sensitivity to medium and high events. This was observed either when comparing the demanding condition to a control (Stroop), or when modelling linearly the difficulty/performance of each depleting task (N-Back). We provide converging evidence that cognitive exertion affects the subsequent appraisal of one’s own and likewise others’ pain.

## Introduction

Identifying and assessing the intensity of pain experienced by others is a crucial ability. This skill is relevant in many clinical and private contexts^1–5^, but especially for caregivers (physicians, nurses, lay individuals in full-time charge of family members, etc.) who have to continuosly monitor others’ pain and might be subjected to fatigue, poor sleep and long/irregular work shifts that may overload of their cognitive resources. However, it is still unclear how extensive mental exhaustion might interfere with the assessment of others’ pain.

In this study we sought to investigate pain assessment under the lens of the well-known “ego depletion” task^6,7^. This paradigm assumes that continuous exertion of self-control exhausts one’s own cognitive resources, impacting subsequent use of similar regulatory processes in an active task, decision-making or even the reaction to an aversive event. Importantly, these effects are held to occur across domains, thus suggesting that self-control rests in part on common regulatory abilities^6–8^. In this perspective, few studies tested the effect played by cognitive exertion in first-hand pain, to get a glimpse on the role played by one’s resources in the subsequent appraisal and reaction to nociceptive stimuli. However, whereas one study revealed that being engaged in a highly demanding Stroop task decreased the sensitivity to subsequent intense stimulations^9^, others found instead increased sensitivity to mild noxious events^10,11^. Such mixed findings beg for a systematic investigation of after-effects of cognitive exertion on different pain levels, and across different depleting tasks, to account also for concerns about reliability of such paradigm which have recently been put forward by the research community^12,13^.

Furthermore, it is unclear whether cognitive exertion would impact also the assessment of pain of others, and whether this influence is similar to that observed one’s own experiences. Seminal models from social psychology/neuroscience suggest that others’ suffering is partially processed in an *embodied* fashion, i.e., by recruiting the same mechanisms underlying one’s own first-hand experiences^14–18^. Indeed, analgesic manipulations (e.g., acetaminophen, hypnosis, placebo) can similarly influence the sensitivity to self and others’ pain^19–21^. Furthermore, neuroimaging studies suggest that self and others’ pain share a partly-common neuronal representation in a widespread network including the middle cingulate cortex^19,22,23^, a brain region held to play a key role in the regulation of one’s pain responses^11,24^ (but see^25^). Hence, it is reasonable to assume that such regulatory mechanisms might equally impact the representation of one’s and others’ pain.

An independent line of research has investigated the role played by cognitive resources in decoding emotional faces. This was chiefly achieved by engaging participants in emotion labelling concurrently to a working memory paradigm requiring high attentional load. The majority of studies report that the processing of emotional states (especially negative) is affected by the task^26–28^. Furthermore, neuroimaging investigations has systematically associated the processing of emotional expressions with brain structures such as the amygdala and fusiform gyrus (see^29^ as meta-analysis), but also found that the activity of these regions was significantly reduced when attentional resources where limited by a competing task^30,31^. These studies suggest that emotion recognition is tightly intertwined with higher-order cognitive processes, something that could potentially generalize also to the case of painful expressions. However, this research is grounded in paradigms engaging working memory tasks concurrently to the processing of facial expressions, thus opening the question if the effects observed underlie divided attention (reflecting the cost of being engaged in two activities at once), or cognitive exertion (whose effects instead linger also after the task’s completion).

**Figure 1:**
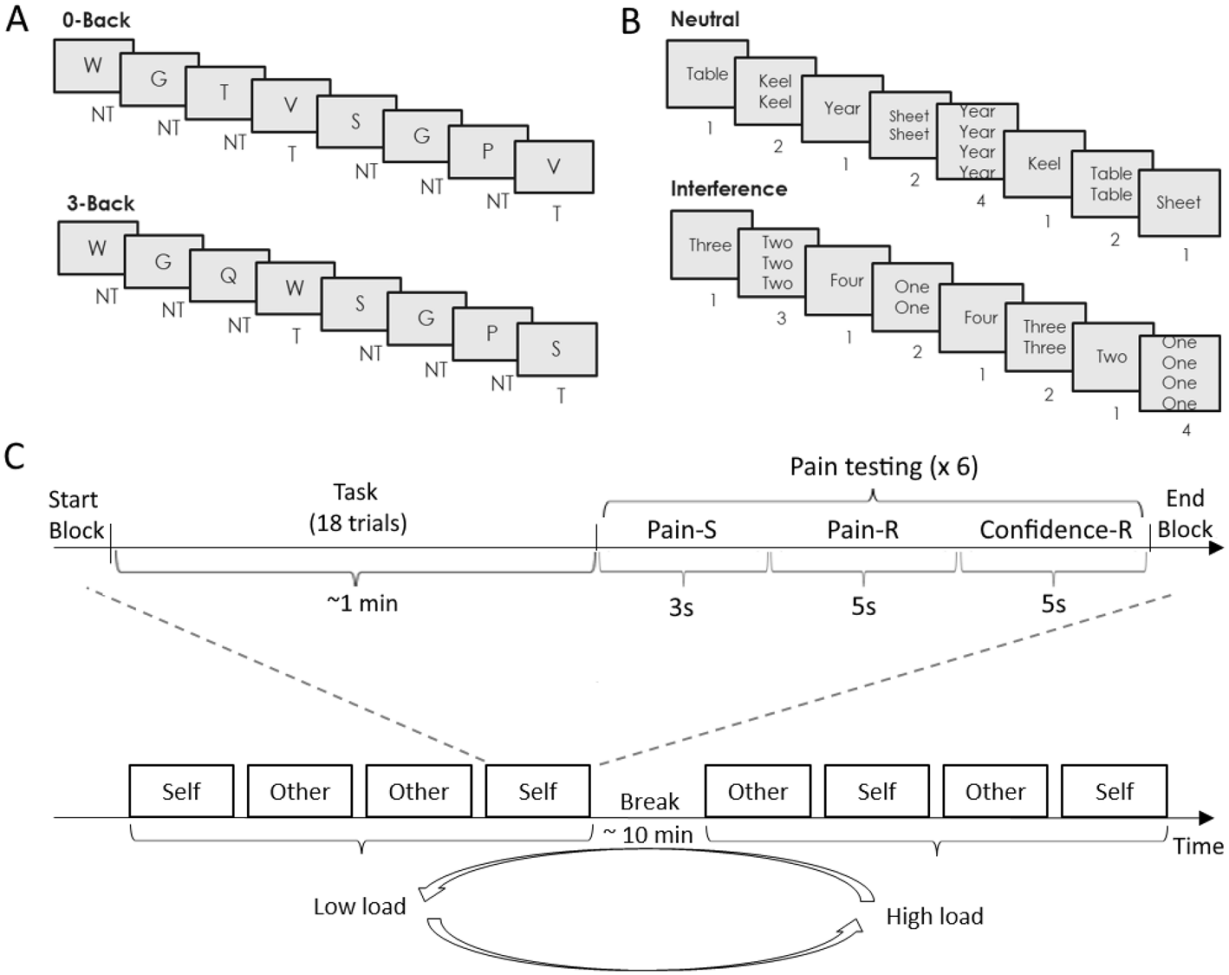
Experiments procedure including tasks, for the experiment 1 the N-back task (**A**) and for the experiment 2 the Stroop task (**B**). Each participant performed four blocks of a High load condition (cognitive exertion) and four blocks of a Low load condition (baseline), presented in a pseudorandom order with a break in between of approximately 10 min. Each block was followed by six pain stimulations of 3 randomized intensity levels (Low, Medium, and High), two intensities per each level, delivered to test self pain (through nociceptive laser stimulations) or other’s pain (through video-clips). After each stimulation trial, participants were asked to rate pain intensity and pain confidence. Overall, the experiment lasted about 1 h and half (**C**). For the Stroop task, words were displayed in French: Table = Table; Keel = Quille; Year = An; Sheet = Drap. NT = No Target; T = Target; Pain-S = Pain Stimulation; Pain-R = Pain Rating; Confidence-R = Confidence Rating.

Here, we engaged participants in one of two demanding paradigms, involving either working memory (Experiment 1: N-Back task) or inhibition (Experiment 2: Stroop task), to investigate the effects of cognitive exertion on the subsequent assessment of one’s and others’ pain (Fig. 1). Although the two tasks underlie different aspects of human executive function, they should both tap a broad regulatory process held to underlie depletion effects. Based on previous research, we expected both demanding tasks to influence intensity ratings of self-pain, albeit with unascertained direction^9,10^. The critical question was whether the manipulation would influence also the pain perceived in others in similar fashion to first-hand experiences (as predicted by *embodied* accounts), or simply by making individuals less sensitive to face-related information.

## Experiment 1

### Methods

#### Participants

Recruitment took place through advertisements posted at the University of Geneva buildings and online platforms. We excluded from our experiment all individuals with the following characteristics: history of alcohol or drug abuse, history of neurological or psychiatric illness, history of chronic pain, fever or an ongoing acute medical condition and extreme dark skin colour (because radiation absorption of dark skin is higher for the wavelength of the laser device used for nociceptive stimulations^32,33^). No recruitment constraints were applied to gender or age. None of the participants reported using medication. They declared good health and typical cognitive proficiency, they had good (or corrected) visual acuity, and were naïve to the purpose of the study.

Previous studies reported Stroop after-effects on self-pain in an overall cohort of N = 24^10^, after excluding those individuals who were not susceptible to the task manipulation (who found the main task easier than its associated control)^10^. The same sample size was also sufficiently powerful to show effects on an N-Back task on concurrent pain, after excluding those individuals who were insensitive to the nociceptive stimulation^34^. As such, we sought to acquire a cohort of similar size which, in addition to the criteria mentioned above, were also filtered for pain insensitivity and task susceptibility. Hence, we recruited a total of 36 participants. Ten were subsequently excluded due to lack of sensitivity to the nociceptive stimulation, whereas 1 was excluded due to the low susceptibility to the task manipulation (see Data Processing subsection below for more details). This led to a final sample of N = 25 (14 females; aged 19 to 37, Mean = 25.16, SD = 5.03 years). This study was approved by the local ethical committee (*Commission Cantonale d'Éthique de la Recherche* of Geneva, protocol code: CCER N. 2019-01355) and conducted according to the Declaration of Helsinki.

#### Experimental set-up

In the present study, volunteers assessed their pain sensitivity and judgments of others’ pain after a low or high cognitive demanding N-Back task. Within this framework, we systematically modulated workload (factor Load: low vs high), type of pain (factor Type: self vs other) and pain intensity (factor Intensity: low, medium, high).

##### N-Back task

We administered an N-Back task^35^, which was previously used successfully to evaluate the impact of distraction on pain sensitivity^34^. Participants monitored a series of stimuli written in black appearing one at a time on a grey screen (Fig. 1A). They were required to identify if the stimulus presented was the same as the one presented *n* previous items by pressing one button (‘yes’) and if not by pressing a different button (‘no’). In the current study, *n* was set to be 3 (high cognitive load condition) or 0 (low cognitive load condition). In the 3-Back condition, participants were required to constantly update the target letter, by retrieving from working memory the one letter occurring 3 items before. For instance, if we pretend a sequence of letters "RGSR", correct answer to the last would be "yes", as the same letter occurred also three trials before in the sequence "RSGP" but in this case answer is "no". As control, we implemented a low cognitive load condition, in which participants were asked to respond only on the trial that they were currently processing, by pressing “yes” whenever a predefined stimulus (“V” letter) would be presented. In each block, a total of 18 letter stimuli were presented one at a time with 4 target-hits.

Each letter was presented for 500 ms, followed by an inter-trial interval (ITI). The ITI between letters of the 0-Back condition was fixed at 700 ms. Instead, for the 3-Back condition to reduce within-participant variation, the ITI was adapted based on participants’ performance in the previous block (i.e., corresponding to 18 previous trials). This is roughly comparable to a previous study with a similar design (adaptation based on previous 15 trials^34^), although in that case, the authors tested pain concurrently to the N-Back, and therefore had no need to divide the experimental session into small blocks^34^. Target sensitivity was assessed with the non-parametric signal detection measure A’^36,37^. This parameter is computed from a pair of hit and false alarm rates^34^: a value near 1 indicates a good discriminability, whereas a value near 0.5 (when hits are equal to false alarm) indicates chance performance. Initial duration of the empty screen was 700 ms. Then based on the previous trial, when discriminability was higher than the targeted level of A’ = 0.75, empty screen interval was reduced, while with a discriminability lower than A’ = 0.75 it was increased.

Prior to the main experiment, participants carried out a brief training session, in which the order of the blocks was fixed: the first block was 0-Back, the second block was 3-Back in which subsequent adjustments were made after each trial. Instead, in the main experiment, the order of blocks was pseudorandomized, and difficulty of the task was calibrated by adapting the interval for each participants’ performance to the previous 3-Back block. Thirteen participants (7 women) started with two blocks 0-Back condition (low cognitive load) and then two blocks with high cognitive load (combined with 2 “Self” and 2 “Other” in a pseudorandomized order) followed by a break of about 5 min and then the same task sequence was repeated (combined with 2 “Self” and 2 “Other” in a pseudorandomized order). With the same procedure, twelve participants (7 women) started with the 3-Back condition (high cognitive load).

##### Self-pain

Half of the N-Back blocks were followed with painful stimulations on their own body. Self-pain was administered through nociceptive radiant heat stimuli was delivered by an infrared neodymium: yttrium–aluminum-perovskite laser system (Neodinium: Yttrium Aluminium Perovskite; Stimul 1340 El.En®; wavelength 1.34 µm; pulse duration 4 ms, beam diameter 6 mm^2^). At this short wavelength, the skin is highly transparent to laser stimulation, and consequently, the laser pulses directly and selectively activate Aδ and C fibers nociceptive terminals located in the superficial layers of the skin^38,39^. Since these fibers have different conduction velocity, participants experienced a double sensation: an initial pricking pain due to Aδ-fiber stimulation, followed by a C-fiber-related burning pain. Laser pulse was transmitted through an optic fiber of 10-m length, with a diameter of 6 mm by focusing lenses. Laser beam was directed to a rectangular skin area (approximately 4 × 2 cm, main axis mediolateral) on the back of the non-dominant hand. To avoid receptor sensitization, as well as damages from long-term exposure, the laser beam was relocated after each trial within a predefined stimulated skin area (see also^40^).

To ensure protection from adverse effects of the laser, the following procedure were put in place. The experiment was carried out in an ad hoc room, protected with laser safety curtains certified for the wavelength of the stimulator. Furthermore, participants and experimenters wore eye-protection goggles with optical density ≥ 2 at 1.34 µm. Participants were asked to remove all accessories from their non-dominant hand to allow safe stimulation of the skin area.

Prior to the beginning of the experiment, pain stimulus intensity was individually calibrated for each participant based on a random staircase thresholding session^41^ to determine stimulations eliciting three levels of pain that were then used in the main experiment. Each trial started with a flash appearing on the screen for 3 s indicating that the stimulation was about to come, while the laser was preparing to deliver the correct amount of energy. After each stimulation, participants answered on a 10-point visual analogue scale ranging from not at all painful (0) to worst pain imaginable (10). Extreme manikins of the arousal scale of the Self-Assessment Manikin (SAM) were displayed at the extremities of the scale for assessing pain intensity as an economical and straightforward cue^42^. We also included numbers under the rating line, coloured from green (0) to red (10), to visually help participants choosing their answer. In this context, participants were instructed to consider the landmark “4” on the scale as the ideal point where they started to feel the stimulation as painful. The rating was self-paced with no time restrictions. The calibration session lasted about 15 min. At the end of this session, we obtained a pain curve based on which we would then select three different energies that corresponded to three different levels of pain for each participant for low pain (LP), medium pain (MP) and high pain (HP) intensity respectively (medians of about 2, 4 and 6 on the pain rating scale).

In the main experiment, after each corresponding N-Back block, participants were exposed to six stimulation-trials in random order. These were characterized by 2 Low, 2 Medium and 2 High pain stimulations. Each trial started with a flash appearing on the screen for 3 s indicating that a stimulation was about to come. The nociceptive stimulus was followed by a black screen lasting for 1 s and then the visual analogue scale for pain judgement. This was identical to the one used in the calibration session, except for the absence of intermediate tick lines, and a time constraint of 5 s for providing a response.

After the pain intensity rating, a 1 s of black screen was presented, followed by a second rating scale. This was a pain metacognition scale probing for participants’ confidence about their previous rating. In particular, participants had to express their confidence about their previous pain judgment on a 10-point visual analogue scale ranging from no confidence at all (0) to completely confident (10). Extreme manikins of the dominance scale of the Self-Assessment Manikin (SAM) were included at the extremities of the metacognition scale^42^. As for the case of pain intensity ratings, participants had 5 s to provide a rating. This second scale was introduced to assess a secondary hypothesis that cognitive exertion could influence, not only participants’ pain assessment, but also the degree of which they were aware of such potential influence.

Following the confidence assessment, a black screen appeared for a random inter-trial interval ranging between 2.5 and 4.5 s with steps of 200 ms. Overall, each block, and subsequent pain stimulations, lasted about 3 min.

##### Other-pain

The remaining half of the blocks were followed by saw video-clips describing pain in others. For this purpose, we used stimuli taken from a database of videos of patients faces, which had spontaneous (non-simulated) expressions of pain^43^. Thirty videos were cut to last 3 s and to show the most salient expressions. For video piloting, 21 independent participants rated these short videos online (14 women; age 22 to 35, Mean = 27.6, SD = 4.7). For the purpose of this experiment, we selected 24 videos based on median ratings of the piloting that could match the three levels of self-pain (8 videos per each level of pain), one characterized by low (3 women, 5 men; Mean = 0.58, SD = 0.50), one characterized by medium (4 women, 4 men; Mean = 3.12, SD = 0.60) and one characterized by high (4 women, 4 men; Mean = 4.70, SD = 0.49) level of painful expression. Furthermore, there was high agreement between the 21 raters in their assessment of the videos of each pain level, as shown by the average inter-rater-correlation (low pain, Spearman’s *ρ* = 0.40 [95% CI: 0.15, 0.67]; medium, *ρ* = 0.50 [0.11, 0.70]; high, *ρ* = 0.65 [0.40, 0.82]) and Cronbach’s α (low, α = 0.81 [0.61, 0.89]; medium, α = 0.91 [0.79, 0.96]; high, α = 0.93 [0.85, 0.96]). Finally, as the people depicted in the videos were also probed to rate their own pain experience^43^, we could thus check that the videos for the three levels of other’s pain exhibited clear-cut differences in low, medium and high self-reported pain. This insured that the selected clips clearly depicted three distinct intensity levels (Low Pain = LP, Medium Pain = MP, High Pain = HP) from both the point of view of the video-recorded people, and from an independent sample of observers.

##### Procedure

The experiment was conducted under the following procedure. On arrival, participants read carefully and signed the informed written consent, then they were reminded that each laser stimulus would be very fast (only 4 ms) and that they could ask to stop the experiment at any time. All tasks and stimuli were coded, managed and presented with Psychophysics Toolbox Version 3 (PTB-3, http://psychtoolbox.org/), a free set of Matlab R2018b (Mathworks, Natick, MA). The experimental session consisted of two parts: (1) pre-test divided in pain thresholding session and training of the N-Back task, and (2) main experiment. Participants had to use a response box with four buttons in their dominant hand to answer. Two experimenters were always present during the experimental session. One experimenter was responsible for technical checks of the equipment before and during acquisition, including physiological measures, laser inputs and behavioural recordings. The other experimenter was responsible for interacting with participants and delivering painful stimulations by directing the laser beam on participants’ hand. Importantly, the latter experimenter was unaware of the level of pain emitted by the laser.

In the main experiment, each participant performed four blocks representing the 3-Back (cognitive exertion) and four blocks of a 0-Back (baseline), presented in a pseudorandom order (Fig. 1A and C). Each block was followed by the delivery of six pain stimulation trials delivered to either the self (through nociceptive laser stimulations) or the other (through video-clips). This led to a 2 (Task: 3-Back *vs*. 0-Back) × 2 (Type: Self *vs*. Other) structure of each block, within each which are administered pain events of 3 intensity levels (Low, Medium, and High). Overall, the experiment session lasted 1 h and half and, at the end, participants were refunded for their time and effort.

##### Questionnaires’ battery

Following the main experimental session, participants were asked to fill some questionnaires. In addition to the general information sheet (including questions about sex, age and hand dominance), participants filled out the Pain Catastrophizing Scale^44^ (PCS) and the Interpersonal Reactivity Index^45^ (IRI), because we hypothesized that these dimensions of personality might modulate the effects of interests. In the PCS, participants were asked to indicate the degree to which they think and feel when they experience pain using a 0 (not at all) to 4 (all the time) scale. A total score is yielded (ranging from 0 to 52), along with three subscale scores assessing rumination (4 questions), helplessness (6 questions) and magnification (3 questions), which respectively report rumination about pain (e.g. "I can´t stop thinking about how much it hurts"), feeling helpless to manage pain (e.g. "There is nothing I can do to reduce the intensity of my pain") and magnification of pain (e.g. "I´m afraid that something serious might happen").

The IRI measures various aspects of empathy including cognitive and emotional empathy. Participants had to answer 28 statements (7 questions per subscale) using a 5-point scale, ranging from “Does not describe me well” to “Describes me very well”. This measure is divided in four seven-item subscales. The perspective taking (PT) scale reports tendency to spontaneously adopt the psychological point of view of others in daily life (e.g., “I sometimes try to understand my friends better by imagining how things look from their perspective”). The empathic concern (EC) scale assesses the tendency to experience feelings of sympathy and compassion for unfortunate others (e.g., “I often have tender, concerned feelings for people less fortunate than me”). The personal distress (PD) scale taps the tendency to experience distress and discomfort in response to extreme distress in others (e.g., “Being in a tense emotional situation scares me”). The fantasy (FS) scale measures the tendency to imaginatively transpose oneself into fictional situations (e.g., “When I am reading an interesting story or novel, I imagine how I would feel if the events in the story were happening to me”).

#### Data processing

##### Behavioral measures

Despite all 36 participants exhibited good sensitivity to pain during the calibration phase, effects of habituation/desensitization might occur in the main experiment. For this reason, we inspected the average rating of the most intense laser stimulation following those blocks which were not held to induce cognitive exhaustion (i.e., 0-Back). We did not include the difficult condition (3-back) as in those blocks sensitivity could have been dampened due to the main manipulation. For ten participants, rating was < 4 on the 10 points intensity scale, and were therefore excluded.

**Figure 2:**
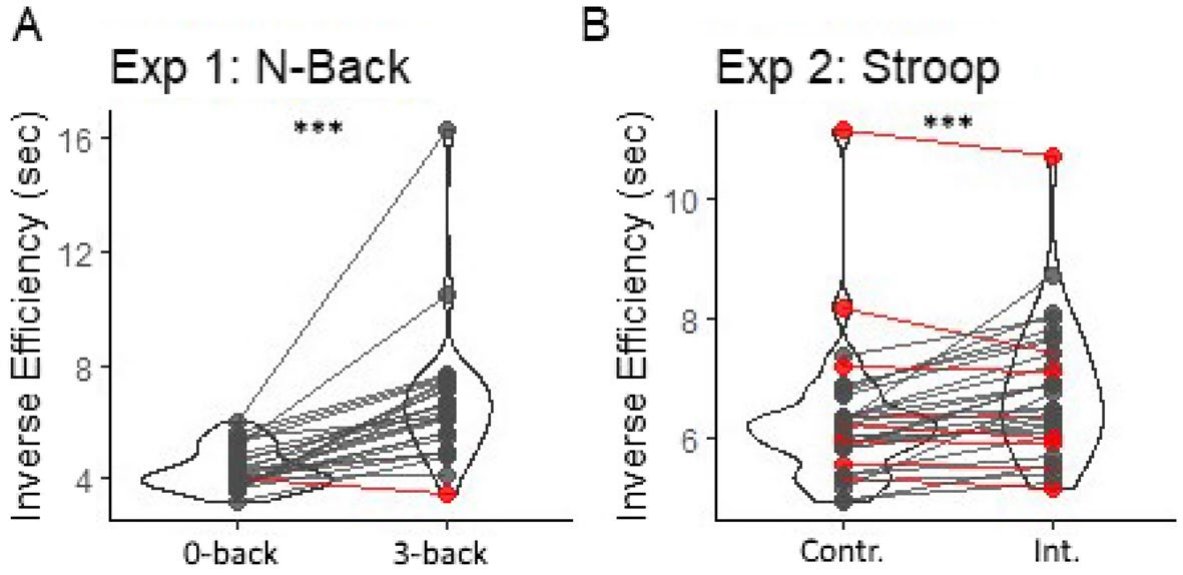
Violin plots and individual inverse efficiency data describing the difference between the two main conditions in (**A**) the N-Back (Exp. 1) and (**B**) Stroop task (Exp. 2). *** p < 0.001 for the effect of task difficulty. Inverse Efficiency scores refer to Response Times of correct responses (sec) penalized proportionally to the number of errors performed in the same condition. Red dots/lines refer to those specific participants for whom the difficult condition was associated with comparable/lower scores than the control condition. *Int.* Stroop Interference. *Contr.* Stroop Control.

We then checked whether participants were susceptible to the N-Back task. To this purpose, we considered all participants who displayed good sensitivity to pain (N = 26). In this analysis, trials with reaction times < 100 ms were removed as potentially reflective anticipatory response unrelated to the task. For each participant, each task and each condition, we calculated the average accuracy, median reaction times of correct responses, and combined Inverse Efficiency Score (IES), with $IES= \frac{Reaction Times}{Accuracy Rate}$^46,47^. This combined measure refers to Reaction Times of correct responses penalized proportionally to the number of errors performed in the same condition (e.g., if a given condition is associated with an accuracy of 90%, IES corresponds to correct Reaction Times multiplied by ~ 10%), and aims at maximizing the information from those cases in which correct Reaction Times and Accuracy are negatively coupled (i.e., conditions eliciting the slowest correct responses being also the least accurate). Omitted responses were considered as errors. After acquisition, all these measures were fed to a paired-sample t-test assessing effects of task difficulty (3-Back *vs*. 0-Back). Then, individual data were used to exclude from all subsequent analyses those participants who were not susceptible to the task (final sample N = 25 see Results and Fig. 2, left subplot).

Having established participants’ susceptibility to the task, we tested its effect on the subsequent pain intensity ratings. This was achieved through a linear mixed model with task condition (3-Back vs 0-Back) and intensity of pain (low, medium, high) as fixed factors. In this case, omitted responses were removed from the analysis. The subject’s identity was modelled as random factor, with random intercept and slope for the two fixed factors and the interaction therefor. Specifically, we ran two models, one for the Self-Pain condition, and the second for the Other-pain conditions. Significance associated with the fixed effects was calculated with a Type III Analysis of Variance using the Satterthwaite approximation of the degrees of freedom, as implemented in the *lmerTest* package^48^ of R (https://cran.r-project.org/) software. In the analysis of pain ratings, effects were considered significant if associated with *p* < 0.05. Each statistical test is also reported in tabular form associated with the correspondent effect size (Cohen’s *d*). Following the main analysis, we also run follow-up models assessing the modulating factor of questionnaires scores. The results of these secondary analyses were considered significant if surviving Bonferroni-correction for multiple comparisons for the number of sub-scores.

##### Physiologic responses

Since it has been shown that cognitive exertion has a negative carryover effects on physical performance^49^ and that physiological states correlate with cognitive fatigue^50^, physiological measures during pain stimulation were also collected to examine possible subsequent effects. Therefore, during the main experiment, we continuously recorded physiological responses to stimuli from their left hand, such as skin conductance (EDA) and heart rate. Detailed information about the analysis of these measures in provided in Supplements.

### Results

#### Task demands

We first assessed whether participants exhibited higher difficulty in the demanding condition. We focused on all participants who displayed adequate sensitivity to pain (N = 26; see “Methods”) and found that, on average, they displayed significant decrease in accuracy in the 3-Back vs. 0-Back condition (*t*_(25)_ = − 8.64, *p* < 0.001; *d* = − 1.73), but also an increase in reaction times of correct responses (*t*_(25)_ = 5.45, *p* < 0.001; *d* = 1.09), and combined inverse efficiency score (IES, *t*_(25)_ = 5.83, *p* < 0.001; *d* = 1.17). This confirms that the 3-Back condition was considered more difficult than its control on the overall population. However, one participant appeared non-susceptible to the manipulation (Fig. 2, left subplot, red line). As mentioned in the Methods section, data from this participant were excluded from subsequent analysis. We thus obtained a final sample of N = 25.

#### Pain ratings

**Figure 3:**
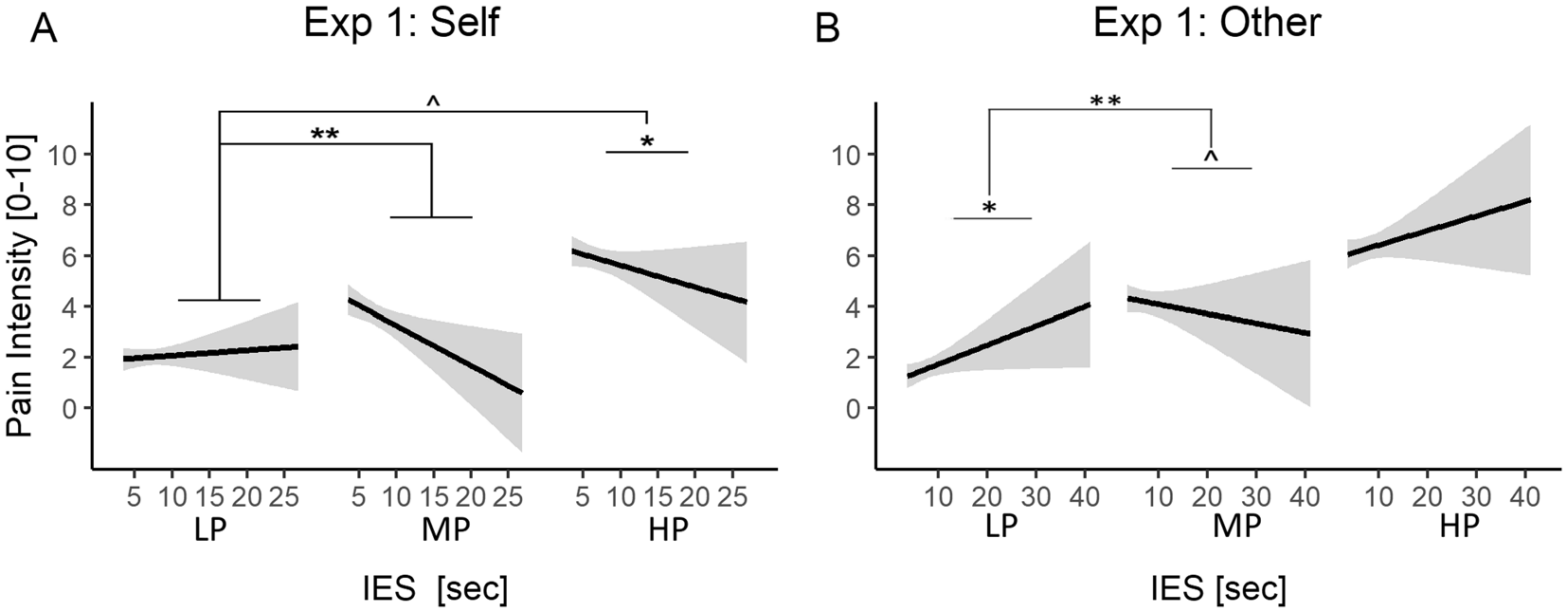
Experiment 1. Analysis of Task Performance. Performance effects on Pain Intensity Stimulation results associated with the N-Back task for Self-Pain **(A)** and Other-Pain data **(B)**. For each pain level, the relationship between IES and pain ratings is described through a linear regression line with 95% confidence interval area. “**”, “*”, “^” refer to simple IES effects for a given pain level, or significant interactions between IES and pain intensity at p < 0.01, p < 0.05, and 0.05 < p < 0.1, respectively. *IES* inverse efficiency score, *LP* low pain, *MP* medium pain, *HP* high pain.

We then assessed whether sensitivity to nociceptive stimulation on one’s own body was influenced by the preceding task. Table 1 reports results associated with pain intensity ratings, and revealed a significant main effect of stimulation *Intensity*, confirming that participants rated both MP and HP as more painful than the LP level. No modulation of *Task* was observed, neither as a main effect, nor in interaction with the *Intensity.* We also checked whether participants’ sensitivity to one’s pain changed as function of performance of the preceding 3-Back condition. This was achieved by modelling participants’ inverse efficiency score (IES, see Methods) from the previous block as a between-subjects continuous predictor. We chose this measure as it represents an efficient combination of correct Response Times and Accuracy values, which prevents us from running two separate analyses. Table 2 and Fig. 3A report results of the main analysis, confirming the main effect of stimulation *Intensity*, but also describing a significant IES*Intensity interaction. In particular, the interaction reveals that blocks associated with poorer performance led to significant decreased sensitivity to MP (*vs*. LP) stimulations, meaning the poorer the performance (high IES) the higher the subsequent hypoalgesia. A similar interaction was observed also for HP (*vs*. LP), although only at a marginally significant level (Table 2). Finally, as the 3-Back task was adjusted online to stabilize performance across blocks (see “Methods”), IES describes only part of the inter-individual variability in performance, whereas the remaining part of the information is provided by the difficulty parameter (the inter-trial interval, ITI, see “Methods”) of the task and how they changed from block to block. We therefore also modelled responses as function of the median ITI from the preceding block. However, such parameter did not affect participants’ sensitivity to pain, neither as main effect nor in interaction with intensity (see Table 2).

**Table 1:** Experiment 1: Analysis of Task-effects on pain.

	Self pain		Other-pain	
	t	*d*	t	*d*
Intercept	9.43***	**1.86**	5.00****	**1.00**
Task	− 1.75	− 0.17	0.37	0.06
Intensity MP	4.72***	**0.91**	9.29***	**1.69**
Intensity HP	10.53***	**2.11**	15.21***	**2.92**
Task*MP	0.73	0.11	− 0.54	− 0.08
Task*HP	0.30	0.04	0.30	0.04

We report the t-values and effect sizes (d) associated with parameter estimates from linear mixed model analyses testing effects of task on Self and Others’ pain. Significant effects are highlighted based on the corresponding p-value.

*Task* contrast 3-back vs. 0-back, *MP* contrast medium pain vs. low pain, *HP* contrast high pain vs. low pain.

***, **, * indicate parameters significantly different from 0 at p < 0.001, p < 0.01, p < 0.05, respectively.

**Table 2:** Experiment 1: Analysis of task performance.

	Self-pain: *IES*		Other-pain: *IES*		Other-pain: *ITI*	
	*t*	*d*	*t*	*d*	*t*	*d*
*Intercept*	9.33***	**1.85**	6.11***	**1.22**	6.22***	**1.21**
*Intensity MP*	6.17***	**1.07**	9.18***	**1.57**	9.47***	**1.43**
*Intensity HP*	10.81***	**2.23**	13.43***	**2.81**	14.02***	**2.84**
*Covariate*	0.94	0.09	1.90	0.19	1.50	0.10
Cov.*MP	− 2.81**	**− 0.20**	− 2.89**	**− 0.24**	− 2.50*	**− 0.17**
Cov.*HP	− 1.76^	− 0.15	− 0.95	− 0.10	− 1.73	− 0.12

We report the t-values and effect sizes (d) associated with parameter estimates from linear mixed model analyses testing effects of task performance and difficulty on Self and Others’ pain. Significant effects are highlighted based on the corresponding p-value.

*MP* contrast medium pain vs. low pain, *HP* contrast high pain vs. low pain. *Cov.* covariate of interest (IES, ITI).

*IES* = *Inverse Efficiency Score; ITI* = *Inter-trial Interval.*

***, **, * indicate parameters significantly different from 0 at p < 0.001, p < 0.01, p < 0.05, respectively.

^ indicate effect at the marginal (0.05 < p < 0.1).

Next, we analysed pain ratings associated with videos of patients displaying painful/painless facial expressions. Results are reported in Table 1 and reveal a significant main effect of pain *Intensity*, confirming that participants rated both MP and HP as more painful than the LP level. No modulation of *Task* was observed, neither as a main effect, nor in interaction with *Intensity*. We also checked whether participants’ sensitivity to one’s pain changed as function of performance of the preceding 3-Back condition, as described by both IES and ITI values. Results are displayed in Table 2 and Fig. 3B, confirming the main effect of stimulation *Intensity*, but also describing significant IES*Intensity and ITI*Intensity interactions. In particular, blocks associated with poorer performance led to significant decreased sensitivity to MP (*vs*. LP) stimulations, for both IES and ITI (Table 2 and Fig. 3).

#### Questionnaire scores

We repeated the analyses of pain ratings by including scores of relevant questionnaires of interest as covariates, in order to identify potential determinants of inter-individual differences in our effects. Specifically, we considered the Pain Catastrophizing Scale^44^, which is expected to tap key components involving attention and cognitive control of pain. As this questionnaire contains three subscales of interest (Rumination, Helplessness and Magnification), each of which could lead to plausible relevant effects, we report effects if associated with an α ≤ 0.0166 (corresponding to Bonferroni-corrected α ≤ 0.05 for three multiple tests). We found no effects associated in any of the three sub-scores in any of the measures of Self- and Other-Pain.

**Figure 4:**
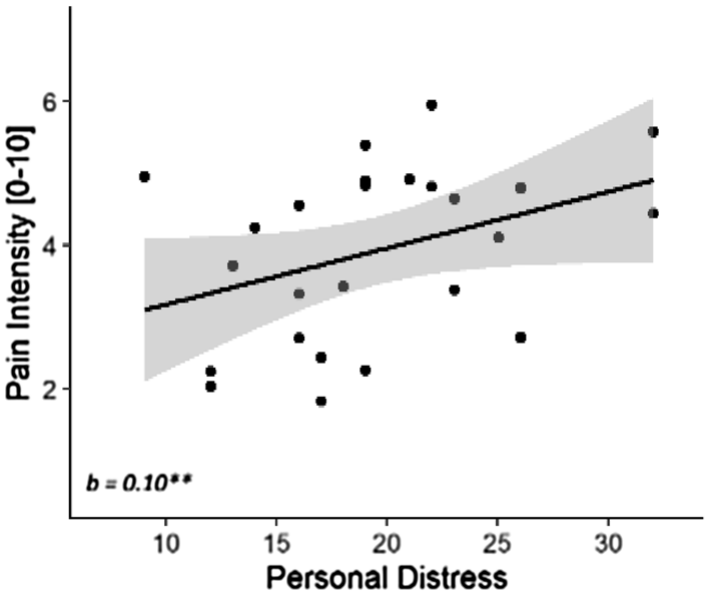
Experiment 1. Personal distress effect on other-pain data. Individuals with higher scores on the Personal Distress subscale of IRI were more prone to rate others’ expressions as more painful. The relationship is described in terms of a linear regression line, associated with 95% confidence intervals area and data-points.

We also tested whether scores of the Interpersonal Reactivity Index^45^, a standardized questionnaire of individual empathic traits, could influence the Other-Pain effects. As this questionnaire contains four subscales of interest (Personal Distress, Perspective Taking, Empathic Concern, Fantasy), we report effects if associated with an α ≤ 0.0125 (Bonferroni-corrected for four tests) for the Other-Pain condition. We found only a significant main effect of *Personal Distress* in the analysis of Pain intensity ratings, suggesting that individuals with higher scores are more prone to rate others’ expressions as more painful (*t*_(29.04)_ = 3.20, *p* = 0.003; *d* = 0.59; see Fig. 4). No other main/interaction effects were associated with any of the questionnaire scores.

#### Additional measures

Supplementary Results report also details about the effects associated with physiological responses, and confidence ratings. Briefly, in the Self-pain condition all physiological measures (skin conductance response and heart rate variability) were significantly associated with a main effect of stimulation *Intensity*, reflecting a progressing increase of response, the stronger energy levels. We found neither an effect of *Task*, nor a modulation of participants’ performance (IES, ITI) on pain response. Instead, no significant effect was associated with the Other-pain condition. Finally, the analysis of confidence ratings revealed no significant effects.

## Experiment 2

Experiment 2 was carried out as a conceptual replication of Experiment 1, aimed at verifying whether the effects observed could be found also when manipulating cognitive exhaustion through another paradigm. For this reason, we run a new study which was identical to Experiment 1, except that the N-Back task was replaced with Stroop.

### Methods

Unless stated otherwise, recruitment criteria, experimental set-up, procedure and data analyses were identical to those used in Experiment 1.

#### Participants

We recruited a total of 39 participants, six of which were subsequently excluded due to falling asleep during data acquisition (one participant) or lack of sensitivity to the nociceptive stimulation (five participants). We further excluded 8 participants who were not susceptible to the task manipulation. This led to a final sample of 25 individuals (14 females; aged 18 to 36, Mean = 23.52, SD = 4.28 years). The exclusion/inclusion criteria were the same as in Experiment 1. In addition, as Experiment 2 involved verbal material, we recruited only French native speakers. The sample size was selected a priori to be identical to that from Experiment 1. None of the participants recruited for Experiment 2 took previously part to Experiment 1.

#### Stroop task

In Experiment 2, N-Back blocks were replaced by Stroop sessions. This task has been extensively used in experimental psychology to modulate participants’ level of cognitive exertion and control^51^. Furthermore, it has been repeatedly shown to influence sensitivity to concurrent and subsequent pain response^9–11,52–55^. In the present study, we used the same version of the Stroop-task used in our previous research on the role of cognitive exertion aftereffects and pain^10,11,54,56^. In particular, each participant performed neutral and interference conditions of the numerical Stroop task^57^. Participants were informed that they would see sets of one to four identical words written in black presented vertically on a grey screen. They were asked to count how many times a word was presented on the screen using buttons of the response box as quickly and accurately as possible, with a strong emphasis placed on not sacrificing accuracy for speed. Moreover, participants were asked not to use strategies to make the task easier (like blurry words on the screen when you almost close your eyes). In the interference condition, words stimuli were number words ‘one’, ‘two’, ‘three’ and ‘four’—presented in French (‘un’, ‘deux’, ‘trois’, ‘quatre’) and they were always incongruent stimuli (word meaning and number of repetitions were different). In the neutral condition, words stimuli were neutral words matched in length with the number words: ‘an’, ‘drap’, ‘table’ and ‘quille’, corresponding in English to ‘year’, ‘sheet’, ‘table’ and ‘keel’. All words were presented in capital letters. Trials started with a fixation cross (1000 ms) followed by a display with words to count which stayed on the screen until participants gave their response but no more than 1250 ms.

Prior to the main experiment, participants carried out a brief training session, in which they were administered 18 practice trials with neutral condition. During the training they received correctness feedback and were also informed to answer more quickly if they did not provide an answer within the allotted time. Feedback appeared for 2500 ms. In the main experiment, no feedback and speed-instruction were presented (Fig. 1B and C). In each block, a total of 18 stimuli were presented. Furthermore, to control for order effects half of participants (n = 13, 6 women) were administered first four blocks with neutral condition (2 “Self” and 2 “Other” in pseudorandomized order), followed by a break of about 10 min and then four blocks with interference condition (2 “Self” and 2 “Other” in pseudorandomized order). Half of participants instead had the interference condition first (n = 12, 9 women).

### Results

#### Task demands

We first assessed whether participants exhibited higher difficulty in the demanding condition. We focused on all participants who displayed adequate sensitivity to pain (N = 33; see “Methods”) and found that, on average, the Stroop Interference was associated with higher reaction times of correct responses than the control neural condition (*t*_(32)_ = 5.53, *p* < 0.001; *d* = 0.98). Similar effects were observed for the inverse efficiency score (IES, *t*_(32)_ = 4.41, *p* < 0.001; *d* = 0.78). However, 8 participants appeared non-susceptible to the manipulation (Fig. 2, right subplot, red lines). As in Experiment 1, the data from these 8 participants were excluded from subsequent analysis, thus leading to a final sample of N = 25.

#### Pain ratings

**Figure 5:**
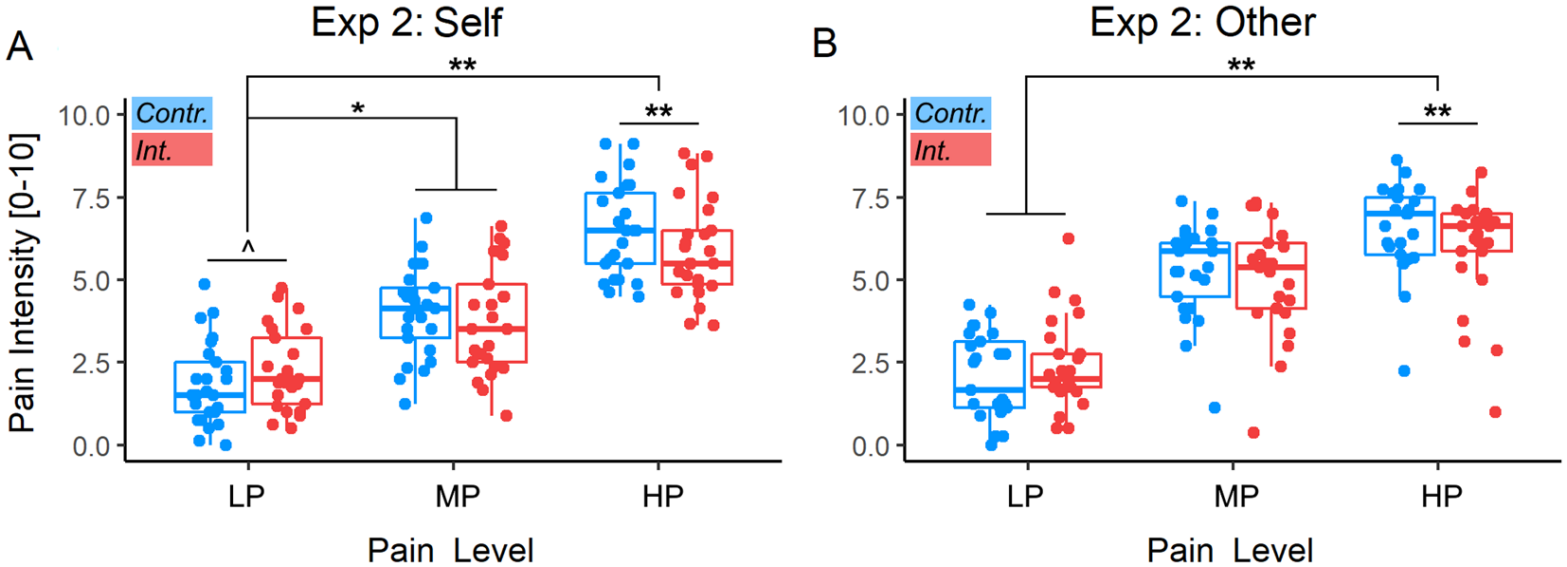
Experiment 2. Analysis of Task-effects on pain. Pain intensity Stimulation for Self-pain (**A**) and Other-pain data (**B**). Red boxplots and data refer to nociceptive stimulations occurring after the Interference [Int.] Stroop condition, whereas blue boxplots/data refer to stimulations following the easy Control [Contr.]. Box plots are described in terms of median (horizontal middle line), interquartile range (box edges), and overall range of non-outlier data (whiskers). Dots refer to individual average values associated to each condition, and are considered outliers if exceeding 1.5 inter-quartile ranges from the median. **, *, ^ refer to significant task main effects for a given pain stimulation level, or to significant interactions between Task and pain intensity at p < 0.01, p < 0.05, and 0.05 < p < 0.1 respectively. *LP* low pain, *MP* medium pain, *HP* high pain.

We then assessed whether sensitivity to nociceptive stimulation on one’s own body was influenced by the preceding task. Table 3 and Fig. 5A report results associated with pain intensity ratings, and revealed a significant main effect of stimulation *Intensity*, confirming that participants rated both MP and HP as more painful than the LP level. We also found that participants’ ratings were influenced by the preceding task, in the shape of a Task*Intensity interaction. We further explored this interaction through simpler models testing effects of *Task* for each stimulation intensity level. The interaction was explainable by a significant decreased response to HP following Stroop interference *vs*. control (*t* = − 2.79, *p* ≤ 0.006). In addition, there was a marginal increase in pain ratings for LP (*t*_(22.56)_ = 1.80, *p* = 0.085; *d* = 0.38), in line with what already observed in previous studies^10,11^. We also checked whether participants’ sensitivity to one’s pain changed as function of performance of the preceding Stroop condition, by modelling participants’ IES from the previous block as a between-subjects continuous predictor. Results are displayed in Table 4, confirming the main effect of stimulation *Intensity*, but providing no evidence of an effect of IES, neither as main effect or in interaction with other variables.

**Table 3:** Experiment 2: Analysis of task-effects on pain.

	Self-pain		Other-pain	
	*t*	*d*	*t*	*d*
Intercept	7.15***	**1.43**	8.04***	**1.62**
Task	1.56	0.27	1.42	0.28
Intensity MP	7.71***	**1.45**	12.47***	**2.30**
Intensity HP	14.95***	**2.92**	15.65***	**2.94**
Task*MP	− 2.00*	**− 0.19**	− 1.66	− 0.34
Task*HP	− 3.09**	**− 0.32**	− 2.82**	**− 0.40**

We report the t-values and effect sizes (d) associated with parameter estimates from linear mixed model analyses testing effects of task on Self and Others’ pain. Significant effects are highlighted based on the corresponding p-value.

*Task* contrast stroop interference vs. neutral, *MP* contrast medium pain vs. low pain, *HP* contrast high pain vs. low pain.

***, **, * indicate parameters significantly different from 0 at p < 0.001, p < 0.01, p < 0.05, respectively.

**Table 4:** Experiment 2: Analysis of task performance.

	Self-pain: *IES*		Other-pain: *IES*	
	*t*	d	*t*	d
*Intercept*	9.16***	**1.87**	8.91***	**1.83**
*Intensity MP*	5.58***	**1.06**	8.11***	**1.63**
*Intensity HP*	12.01***	**2.51**	9.83***	**2.02**
*IES*	0.90	0.10	− 1.20	− 0.14
IES*MP	− 0.74	− 0.07	− 0.51	− 0.06
IES*HP	0.09	0.01	− 0.15	− 0.02

We report the t-values and effect sizes (d) associated with parameter estimates from linear mixed model analyses testing effects of task performance and difficulty on Self and Others’ pain. Significant effects are highlighted based on the corresponding p-value.

*MP* contrast medium pain vs. low pain, *HP* contrast high pain vs. low pain, *IES* inverse efficiency score.

***, **, * indicate parameters significantly different from 0 at p < 0.001, p < 0.01, p < 0.05, respectively.

Next, we analysed pain ratings associated with videos of patients displaying painful/painless facial expressions. Results are reported in Table 3 and Fig. 5B and reveal a significant main effect of pain *Intensity*. As for the case of the Self-Pain ratings, we observed also Task*Intensity interaction. We further explored this interaction through simpler models testing effects of *Task* in each pain level, which was explained by a significant decreased response to HP following Stroop interference *vs*. control (*t*_(67.11)_ = − 2.97, *p* = 0.004; *d* = − 0.36; Fig. 3B). Finally, we also checked whether participants’ sensitivity to other’s expressions changed as function of performance of the preceding depleting task, but found no effect associated with IES.

#### Additional measures

As for Experiment 1, we repeated the analyses of pain ratings by including the scores of the Pain Catastrophizing Scale^44^, and Interpersonal Reactivity Index^45^, as predictor. No significant effect was found. Supplementary Results report also details about the effects associated with physiological responses, and confidence ratings. Briefly, in the Self-pain condition all physiological measures (skin conductance response and heart rate variability) were significantly associated with a main effect of stimulation *Intensity*, meaning that independently from the task the three levels of pain were significantly different. Furthermore, we found that physiological measures were also modulated by the preceding task, in the shape of a Task*Intensity interaction (for skin conductance level) or a IES*Intensity interaction (for hearth rate variability). In both cases, the interaction is explainable in terms of decreased response to HP (vs LP) following a more challenging block. No significant effect in physiological responses was associated with the Other-pain condition. Finally, the analysis of confidence ratings revealed no significant effects.

## Discussion

We tested whether cognitive exertion influences the subsequent evaluation of one’s and other’s pain. For this purpose, we employed two cognitively demanding tasks, a working memory (*Exp. 1*: N-Back)^34^ and an inibition paradigm (*Exp. 2*: Stroop)^26,62,65^, which both decreased the sensitivity to medium/high pain on one’s own body (measured through intensity ratings). These effects were mirrored by a comparable decrease in subsequent assessment of facial expressions, with participants underrating those video-clips displaying the medium/high pain. This was observed either by comparing the effects of demanding condition to those associated with an easier control condition (*Exp. 2*: Stroop), or by modelling linearly the difficulty/performance of each demanding block (*Exp. 1*: N-Back). Finally, and specifically for the Stroop, we also found that cognitive control led to a mild hypersensitization towards self-directed low-pain stimulations (as previously suggested)^10,11^. In both experiments, and in both self- and other-assessments, task modulations on pain sensitivity ranged between a Cohen’s *d* of 0.17 to 0.40, reflecting a magnitude within the small to medium range^58^*.* Overall, our results show that cognitive exertion impacts negatively the assessment others’ suffering, partly mirroring to what happens for first-hand pain experiences.

### Effects on self-pain

Both Stroop and N-Back task influenced self-pain experience as function of stimulation intensity: whereas the sensitivity to MP/HP was decreased following the demanding task, this was not the case for LP stimuli. For the Stroop task, as predicted, we found that HP pain was rated as less intense in the main task, compared to its easier control condition. Instead, for the N-Back task, we found that participants’ performance in each 3-Back block was linearly coupled with the subsequent rating of MP/HP stimulations: specifically, the poorer the performance (high IES) the higher the subsequent hypoalgesia (Fig. 4A). Such effect can be interpreted as a result of cognitive resource restriction (similar to the one observed for the Stroop), under the assumption that poor performance underlies a high level of cognitive fatigue already present during the task, and extending on the subsequent nociceptive stimulations. We believe this to be the most suitable explanation for our data, which also allows to interpret the results from both experiments under a similar theoretical framework.

Previous theories suggest that cognitive regulatory abilities play a major role in downplaying adverse experiences (like those associated with pain), and promote coping reactions. In this perspective, exhausting one’s cognitive resources should weaken these top-down regulatory mechanisms, ultimately increasing the sensitivity to pain^10,11^. This interpretation does not account for our findings, especially the strong hypoalgesic effect for MP/HP observed in both experiments.

To the best of our knowledge, the role played by cognitive exertion on middle/high pain could be explained in two possible ways. First, regulatory processes may be engaged in an active control of low (non-life-threatening) painful situations to allow the pursuit of one’s everyday activity. Instead, when experiencing intense (or enduring) pain, the body may enter in a shock-like state in which suffering responses are automatically dulled^59,60^. Accordingly, the more a noxious stimulus is intense, the more pain regulation might shift from deliberate to automatic, thus leading to hypoalgesia only for medium/high intensities. A second plausible interpretation would imply that cognitive exertion does not influence uniquely top-down regulatory processes, but interferes directly with the representation of pain itself. Indeed, recent accounts suggest that pain experience could be best interpreted within a Bayesian framework, where the brain estimates the (posterior) probability of potential body damage, based on the integration of sensory inputs and prior representations^61–69^. Within this framework, pain should be considered the result of an active inferential process which, under limited resources, could prove challenging for individuals, and lead poorer discrimination between low and high stimulations. This last interpretation would effectively fit all our findings across both experiments.

In the Stroop task, we also observed a marginally increase in sensitivity for LP stimuli. Although it is unclear why this was not observed also for the N-Back task, it is interesting to notice previous studies testing Stroop after-effects document hyperalgesic effects for medium–low noxious stimulations (ratings corresponding to ~ 4 on our scale)^10,11^, whereas others described hypoalgesia for more intense events (~ 6)^9^. In this perspective, by modelling stimulation intensity, we could disambiguate an otherwise mixed literature, and provide clear evidence that the effects of cognitive control after-effects on pain change as function of the intensity of the noxious stimulation.

Finally, it could be argued (at least in principle) that effects associated with the N-Back task do not underlie the same process of cognitive resource restriction observed for the Stroop.

Indeed, according to motivational models of depletion and cognitive fatigue, task subjective difficulty interacts with individual commitment in mobilizing effort^6,54,70^. Hence, a task perceived as too difficult would lead individuals to disengage (high IES), thus impacting only minimally cognitive resources. We believe that this interpretation does not fit entirely our results. Indeed, while it is true that participants who disengaged from the task should be characterized by low proficiency (high IES), they should also be associated with low difficulty (short ITI). Indeed, as the task difficulty was continuously adapted to stabilize as much as possible performance across the population (see Methods), individuals who “gave up” should have rapidly converged towards the easiest task parameters. This was not observed in our study where, in the Self-Pain condition, the ITI did not influence participants’ sensitivity to pain. For this reason, participants’ high IES in our study most likely high level of cognitive exhaustion due to an active engagement in the task.

### Effects on other-pain

In addition to the effects of cognitive exertion of self-pain, our study allowed to assess how these effects generalize to the observation of pain in others. In particular, we built the study in order to test two different (but not mutually-exclusive) predictions. On the one side, the *Embodied* account argues for the presence of shared representation between self and others’ pain^14–18^; in this framework, cognitive exertion is held affect judgments of others’ pain analogously to self-pain. On the other hand, a wide literature pointed to a major the role of attentional resources on facial expression decoding, hinting that a limitation of the former leads to a reduced sensitivity to the latter^26–28,30,31^. Our results provide support to predictions from both lines of research. Indeed, restrictions of one’s cognitive resources did decrease the sensitivity of medium/high painful expressions in both N-Back (as function of task performance and difficulty) and Stroop (relative to a neutral control condition). However, and most importantly, all the task-aftereffects observed in for other’s pain were observed also for the self-pain, with astonishing similarity in terms of the way in which the previous task was modelled (Task factor in Stroop, IES regressor in N-Back) and on the pain levels implicated (HP for Stroop, MP for N-Back). Hence, whatever the effects of each cognitive task on self-pain, similar effects were present also for the observation of others’ painful expression, thus providing strong support to the Embodied account. Interestingly, neuroimaging literature has extensively investigated *Embodied* accounts by quantifying the neural response to others’ pain through fMRI^71,72^ or EEG^73,74^, and its similarity with that of self-pain^21^. One prediction for follow-up research would be that cognitive exertion might decrease the responsiveness of a shared component of one’s and others’ neural response to pain.

### Limitations of the study and conclusive remarks

In our study we excluded participants based on low pain sensitivity or non-susceptibility to the Stroop/N-Back (see “Methods”). These criteria, like other aspects of the experimental protocol, were not subjected to pre-registration prior data acquisition. Furthermore, the non-negligible number of excluded individuals highlights two important limitations. The first is the sub-optimal nature of task manipulation and pain intensity calibration, which might be vulnerable to factors like training, habituation, desensitization, etc. The second is the final sample, which might not be representative of the overall population, but only of those individuals most sensitive to pain and less proficient in the tasks employed. Furthermore, unlike the Stroop task, N-Back aftereffects on pain sensitivity were found only when modelling individual IES as continuous predictor, but not in relation to the a priori designed control 0-Back. We suggest caution interpreting differences between the two experiments, as the two tasks were selected from previous research due to their established efficacy in modulating self-pain experience^10,34^, but they have never been matched with one another in terms of difficulty. However, one possible explanation for this observation could be that memory tasks might be less suited than Stroop for inducing a depletion effect, as suggested by a recent meta-analysis^75^. Cognitive control might be required also in the 0-Back control condition, where participants are asked to only respond to the presentation of a specific target letter (similarly to a “go/no-go” paradigm^76^), thus potentially questioning the effectiveness of this condition as a control for cognitive control effects. Future studies will need to use a more suitable control (e.g., 1-Back).

Notwithstanding these limitations, our study demonstrates that the aftereffect of cognitive exertion on others’ pain judgment decreases the sensitivity towards the most intense stimulations, similarly to what observed in first-hand experiences. Lay individuals and professional caregivers should be aware that high workload and strong cognitive fatigue might alter their diagnostic abilities.

## Supplementary Information

Supplementary Information.

## Data Availability

The data supporting the main findings of this study are available contacting the corresponding author.

## References

[1] Ruben MA, van Osch M, Blanch-Hartigan D (2015). Healthcare providers’ accuracy in assessing patients’ pain: A systematic review. Patient Educ. Couns..

[2] Kappesser J, Williams ACDC, Prkachin KM (2006). Testing two accounts of pain underestimation. Pain.

[3] Bergh I, Sjostrom B (1999). A comparative study of nurses’ and elderly patients’ ratings of pain and pain tolerance. J. Gerontol. Nurs..

[4] Ruben MA, Blanch-Hartigan D, Shipherd JC (2018). To know another’s pain: A meta-analysis of caregivers’ and healthcare providers’ pain assessment accuracy. Ann. Behav. Med..

[5] Rupp T, Delaney KA (2004). Inadequate analgesia in emergency medicine. Ann. Emerg. Med..

[6] Wright RA, Mlynski C (2019). Fatigue determination of inhibitory strength and control: A babe in a bath. Motiv. Sci..

[7] Hagger MS, Wood C, Stiff C, Chatzisarantis NLD (2010). Ego depletion and the strength model of self-control: A meta-analysis. Psychol. Bull..

[8] Halali E, Bereby-Meyer Y, Meiran N (2014). Between self-interest and reciprocity: The social bright side of self-control failure. J. Exp. Psychol. Gen..

[9] Hoegh M, Seminowicz DA, Graven-Nielsen T (2019). Delayed effects of attention on pain sensitivity and conditioned pain modulation. Eur. J. Pain.

[10] Silvestrini N, Rainville P (2013). After-effects of cognitive control on pain. Eur. J. Pain.

[11] Silvestrini N (2020). Distinct fMRI patterns colocalized in the cingulate cortex underlie the after-effects of cognitive control on pain. Neuroimage.

[12] Friese M, Loschelder DD, Gieseler K, Frankenbach J, Inzlicht M (2018). Is ego depletion real? An analysis of arguments. Personal. Soc. Psychol. Rev..

[13] Lin H, Saunders B, Friese M, Evans NJ, Inzlicht M (2020). Strong effort manipulations reduce response caution: A preregistered reinvention of the ego-depletion paradigm. Psychol. Sci..

[14] Bastiaansen JACJ, Thioux M, Keysers C (2009). Evidence for mirror systems in emotions. Philos. Trans. R. Soc. B Biol. Sci..

[15] Bernhardt BC, Singer T (2012). The neural basis of empathy. Annu. Rev. Neurosci..

[16] Caruana F, Jezzini A, Sbriscia-Fioretti B, Rizzolatti G, Gallese V (2011). Emotional and social behaviors elicited by electrical stimulation of the insula in the macaque monkey. Curr. Biol..

[17] Gallese V (2003). The roots of empathy: The shared manifold hypothesis and the neural basis of intersubjectivity. Psychopathology.

[18] Goldman A, de Vignemont F (2009). Is social cognition embodied?. Trends Cogn. Sci..

[19] Braboszcz C, Brandao-Farinelli E, Vuilleumier P (2017). Hypnotic analgesia reduces brain responses to pain seen in others. Sci. Rep..

[20] Mischkowski D, Crocker J, Way BM (2016). From painkiller to empathy killer: acetaminophen (paracetamol) reduces empathy for pain. Soc. Cogn. Affect. Neurosci..

[21] Rütgen M, Seidel E-M, Rieansky I, Lamm C (2015). Reduction of empathy for pain by placebo analgesia suggests functional equivalence of empathy and first-hand emotion experience. J. Neurosci..

[22] Corradi-Dell’Acqua C, Tusche A, Vuilleumier P, Singer T (2016). Cross-modal representations of first-hand and vicarious pain, disgust and fairness in insular and cingulate cortex. Nat. Commun..

[23] Corradi-Dell’Acqua C, Hofstetter C, Vuilleumier P (2011). Felt and seen pain evoke the same local patterns of cortical activity in insular and cingulate cortex. J. Neurosci..

[24] Shackman AJ (2011). The integration of negative affect, pain and cognitive control in the cingulate cortex. Nat. Rev. Neurosci..

[25] Kragel PA (2018). Generalizable representations of pain, cognitive control, and negative emotion in medial frontal cortex. Nat. Neurosci..

[26] Valenti L, Wada Pucci I, Basso Garcia R, Jackson MC, Galera C (2022). Attentional load effects on emotional content in face working memory. Q. J. Exp. Psychol..

[27] Phillips LH, Channon S, Tunstall M, Hedenstrom A, Lyons K (2008). The role of working memory in decoding emotions. Emotion.

[28] Van Dillen LF, Derks B (2012). Working memory load reduces facilitated processing of threatening faces: An ERP study. Emotion.

[29] Liu M, Liu CH, Zheng S, Zhao K, Fu X (2021). Reexamining the neural network involved in perception of facial expression: A meta-analysis. Neurosci. Biobehav. Rev..

[30] Pessoa L, McKenna M, Gutierrez E, Ungerleider LG (2002). Neural processing of emotional faces requires attention. Proc. Natl. Acad. Sci. U. S. A..

[31] Vuilleumier P, Armony JL, Driver J, Dolan RJ (2001). Effects of attention and emotion on face processing in the human brain. Neuron.

[32] Mancini F, Nash T, Iannetti GD, Haggard P (2014). Pain relief by touch: A quantitative approach. Pain.

[33] Baumgärtner U, Cruccu G, Iannetti GD, Treede RD (2005). Laser guns and hot plates. Pain.

[34] Buhle J, Wager TD (2010). Performance-dependent inhibition of pain by an executive working memory task. Pain.

[35] Jonides J (1997). Verbal working memory load affects regional brain activation as measured by PET. J. Cogn. Neurosci..

[36] Stanislaw H, Todorov N (1999). Calculation of signal detection theory measures. Behav. Res. Methods Instrum. Comput..

[37] Snodgrass JG, Corwin J (1988). Pragmatics of measuring recognition memory: Applications to dementia and amnesia. J. Exp. Psychol. Gen..

[38] Plaghki L, Mouraux A (2003). How do we selectively activate skin nociceptors with a high power infrared laser? Physiology and biophysics of laser stimulation. Neurophysiol. Clin..

[39] Iannetti GD, Zambreanu L, Tracey I (2006). Similar nociceptive afferents mediate psychophysical and electrophysiological responses to heat stimulation of glabrous and hairy skin in humans. J. Physiol..

[40] Loued-Khenissi L, Martin-Brevet S, Schumacher L, Corradi-Dell’Acqua C (2022). The effect of uncertainty on pain decisions for self and others. Eur. J. Pain.

[41] Mancini F (2015). Touch inhibits subcortical and cortical nociceptive responses. Pain.

[42] McNeil DW, Brunetti DG (1992). Pain and fear: A bioinformational perspective on responsivity to imagery. Behav. Res. Ther..

[43] Lucey, P., Cohn, J. F., Prkachin, K. M., Solomon, P. E. & Matthews, I. Painful data: The UNBC-McMaster shoulder pain expression archive database. *2011 IEEE Int. Conf. Autom. Face Gesture Recognit. Work. FG 2011* 57–64. 10.1109/FG.2011.5771462 (2011)

[44] Sullivan M (1995). The pain catastrophizing scale. Psychol Assess..

[45] Davis M (1980). A multidimensional approach to individual differences in empathy. JSAS Cat. Sel. Doc. Psychol..

[46] Townsend, J. T. & Ashby, F. G. Methods of Modeling Capacity in Simple Processing Systems. in *Cognitive theory (Vol 3)* (eds. Castellan, J. & Restle, F.) 200–239 (Hillsdale, N.J.: Erlbaum., 1978).

[47] Townsend JT, Ashby FG (1983). Stochastic Modeling of Elementary Psychological Processes.

[48] Kuznetsova A, Brockhoff PB, Christensen RHB (2017). lmerTest Package: Tests in linear mixed effects models. J. Stat. Softw..

[49] Brown DMY (2020). Effects of prior cognitive exertion on physical performance: A systematic review and meta-analysis. Sports Med..

[50] Lee KFA, Gan WS, Christopoulos G (2021). Biomarker-informed machine learning model of cognitive fatigue from a heart rate response perspective. Sensors.

[51] Stroop JR (1935). Studies of interference in serial verbal reactions. J. Exp. Psychol..

[52] Valet M (2004). Distraction modulates connectivity of the cingulo-frontal cortex and the midbrain during pain: An fMRI analysis. Pain.

[53] Marouf R (2014). Reduced pain inhibition is associated with reduced cognitive inhibition in healthy aging. Pain.

[54] Silvestrini N, Gendolla GHE (2019). Affect and cognitive control: Insights from research on effort mobilization. Int. J. Psychophysiol..

[55] Rischer KM (2020). Distraction from pain: The role of selective attention and pain catastrophizing. Eur. J. Pain.

[56] Silvestrini N, Acqua CC-D (2023). Distraction and cognitive control independently impact parietal and prefrontal neural response to pain. Soc. Cogn. Affect. Neurosci..

[57] Bush G (1998). The counting stroop: An interference task specialized for functional neuroimaging—validation study with functional MRI. Hum. Brain Mapp..

[58] Cohen J (1998). Statistical Power Analysis for the Behavioral Sciences.

[59] Kandel E, Schwartz J, Jessell T (2000). Principles of Neural Science.

[60] Bernstein MJ, Claypool HM (2012). Social exclusion and pain sensitivity: Why exclusion sometimes hurts and sometimes numbs. Personal. Soc. Psychol. Bull..

[61] Morrison I, Perini I, Dunham J (2013). Facets and mechanisms of adaptive pain behavior: Predictive regulation and action. Front. Hum. Neurosci..

[62] Tabor A, Thacker MA, Moseley GL, Körding KP (2017). Pain: A statistical account. PLOS Comput. Biol..

[63] Tabor A, Burr C (2019). Bayesian learning models of pain: A call to action. Curr. Opin. Behav. Sci..

[64] Wiech K (2016). Deconstructing the sensation of pain: The influence of cognitive processes on pain perception. Science.

[65] Büchel C, Geuter S, Sprenger C, Eippert F (2014). Placebo analgesia: A predictive coding perspective. Neuron.

[66] Seymour B, Mancini F (2020). Hierarchical models of pain: Inference, information-seeking, and adaptive control. Neuroimage.

[67] Seymour B (2019). Pain: A precision signal for reinforcement learning and control. Neuron.

[68] Sharvit G, Vuilleumier P, Corradi-Dell’Acqua C (2019). Sensory-specific predictive models in the human anterior insula [version 1; referees: 2 approved]. F1000Research.

[69] Ongaro G, Kaptchuk TJ (2019). Symptom perception, placebo effects, and the Bayesian brain. Pain.

[70] Silvestrini N, Corradi-Dell’Acqua C (2022). The impact of pain on subsequent effort and cognitive performance. J. Psychophysiol..

[71] Kogler L, Müller VI, Werminghausen E, Eickhoff SB, Derntl B (2020). Do I feel or do I know? Neuroimaging meta-analyses on the multiple facets of empathy. Cortex.

[72] Lamm C, Decety J, Singer T (2011). Meta-analytic evidence for common and distinct neural networks associated with directly experienced pain and empathy for pain. Neuroimage.

[73] Chen C, Yang CY, Cheng Y (2012). Sensorimotor resonance is an outcome but not a platform to anticipating harm to others. Soc. Neurosci..

[74] Vecchio A, De Pascalis V (2023). ERP indicators of situational empathy pain. Behav. Brain Res..

[75] Carter EC, Kofler LM, Forster DE, McCullough ME (2015). A series of meta-analytic tests of the depletion effect: Self-control does not seem to rely on a limited resource. J. Exp. Psychol. Gen..

[76] Verbruggen F, Logan GD (2008). Automatic and controlled response inhibition: Associative learning in the Go/No-Go and stop-signal paradigms. J. Exp. Psychol. Gen..

